# Finite elements analysis of the temporomandibular joint disc in patients with intra-articular disorders

**DOI:** 10.1186/s12903-020-01074-x

**Published:** 2020-03-30

**Authors:** Linfeng Lai, Chenyao Huang, Fan Zhou, Fujian Xia, Guofeng Xiong

**Affiliations:** Department of Oral Surgery, The Dingling Clinical Institute of Wenzhou Medical University (Wenzhou Central Hospital), Wenzhou Medical University, Wenzhou, Zhejiang People’s Republic of China

**Keywords:** Temporomandibular joint (TMJ), Intra-articular disorders (ID), Finite elements analysis (FEA), Finite elements model (FEM), TMJ disorder (TMD), Friction coefficient

## Abstract

**Background:**

Intra-articular disorders (ID) or anterior and/or medial displacement of the temporomandibular joint disorder (TMJ) disc are the most common form of TMJ dysfunction (TMD). TMD causes changes in the friction coefficient during TMJ movement. Herein, we provided a three-dimensional (3D) finite-elements model (FEM) including the maxilla, disc, and mandible and evaluated the stress distribution with different friction coefficient.

**Methods:**

Fourteen volunteers without TMD and 20 patients with MRI-diagnosed TMD were selected. CT and MRI data were collected to build the 3D FEA model of the mandible and TMJ disc. Stress distribution with different friction coefficient was measured.

**Result:**

In the normal model, stress distribution on the TMJ disc was 2.07 ± 0.17, 1.49 ± 0.14, and 1.41 ± 0.14 MPa with 0.001, 0.3, and 0.4 friction coefficient, respectively. In the TMD model, stress distribution was 3.87 ± 0.15, 7.23 ± 0.22, and 7.77 ± 0.19 MPa respectively.

**Conclusion:**

When the friction coefficient of the side with anterior displacement increased, stress on the disc, condyle and mandible of the opposite side increased. Simultaneously, stress values of the disc, condyle and mandible were higher than those of the normal lateral joint.

## Background

The temporomandibular joint (TMJ) is a bilateral diarthrodial joint of the jaws in the human skeleton [1]This unique joint is the only synovial joint in humans wherein the articulating surfaces are covered by fibrocartilage [2]. TMJ disorder (TMD) is a common condition with an estimated incidence of 20–25% [3, 4]. Anterior and/or medial displacement of the articular disc or intra-articular disorders (ID) are the most common form of TMJ dysfunction. Although exact causes of TMD are complicated and remain inconclusive, ID, microtrauma and intra-articular stress are considered some of the major causes of TMD.

Research on biomechanics of TMJ is limited by the complicated structure of the joint. Traditional biomechanics can cause trauma and have several disadvantages such as non-repeatability and presenting difficulty in comparing different force distributions.

Finite element analysis (FEA) is a numerical method for solving mechanical problems for complex structure. In recent times, FEA has been widely used for research on medical biomechanics, especially that of orthopaedic devices under various loading conditions [5–7]. Owing to imaging difficulties, studies regarding FEA of the TMJ disc are still scarce. In Tanne’s research [8], the 3D FEM of the mandible (including the cortical bone, cancellous bone, articular cartilage, and joint disc) was established by slicing a skull specimen. The joint disc was simulated as a 2-mm-thick tissue covering the surface of skeletal process, which was used to discuss the stress conduction mode of the cranio-maxillary system under stress. Based on a normal human mandibular specimen, CT scanning was used to establish the mandibular model [9]. The stress, strain, deformation, and condylar reaction of the jaw were systematically analysed by this mandibular model. Based on the CT image of a cadaveric head, the 3D FEM of the mandible was established by manual recording. The solid model of the joint disc was established by manual tomography [10–13]. Despite the progress in modeling of TMJ, those model was not capable of accurate soft tissue reconstruction and ignored the influence of the disc in cases with abnormal intra-articular structure. Furthermore, the friction coefficient change in the TMJ of patients with TMD has been rarely considered in previous studies.

In the present study, the CT and MRI images of both volunteers without TMD and TMD patients were collected. Subsequently, three-dimensional (3D) FEA models including the maxilla, disc and mandible were established using 3D data registration technology. This protocol provided data for FEA as well as 3D view of changes in every patient’s TMJ anatomy. Moreover, this protocol could not only aid in diagnosis and treatment of TMDs but also help explore the influence of friction coefficient change in disc displacement.

## Methods

### Patients

From April 2017 to June 2018, healthy volunteers (6 mal and 8 female, age: 18–60 years) and TMD patients (9 male and 11 female, age, 17–60 years) at the Department of Oral Surgery, Central Hospital Affiliated to Wenzhou Medical University were selected. We classified all patients after consultation. The following patients were excluded: (1) those who had undergone any maxillo-facial surgery; (2) those with a history of mental illness; (3) volunteers who were diagnosed with TMD by at least one MRI specialist; and (4) those diagnosed with TMD by two MRI specialists and who had experienced pain with coexisting clicking for > 1 year as well as limitation of mouth opening were included in the study population.

### CT and MRI and data collection

CT scans were performed for all subjects by using 320-channel multidetector scanners (Brilliance, Philips, Netherlands). CT settings were axial 0.625 mm collimation, 120 kVp, auto exposure and table speed of 60 or 32 mm/s.

MRI images were acquired using a 1.5-T scanner (Symphony, Siemens, Olangan, Germany) with a 7.5-cm surface coil. A 3-mm section thickness with a 140-mm field of view and spin-echo multi-section images were used. MRI images were independently evaluated by two experienced oral and maxillofacial radiologists at two different time points. In case of disagreement, final assessment was reached by consensus.

### Construction of FEM of TMJ

A surface mesh model was imported into the FEA software in the initial graphics exchange specification (IGES) format. The volume model was constructed using the bottom-up ‘dot-line-plane-body’ approach. Isotropic, homogeneous and continuous linear elastic materials, which accorded with small deformation conditions, were used. Material constants of each material used in the experiment were extracted from previous studies [14–16] (Table 1).

**Table 1:** Properties of TMJ structures

Tissue	Modulus of elasticity	Poisson ratio
Cortical bone	13,700	0.3
Cancellous bones	7930	0.3
Tooth	18,600	0.31
Disc	30.9	0.4
Bilaminar region	0.49	0.49
Anadesma	68.9	0.45

The maximum masticatory muscle strength of each muscle was calculated by using Koolstra’s formula Li, max = P. A I, where P is an intrinsic strength constant with a value of 0. 37,106 N/m^2^.

Contact was a nonlinear issue, and we could simulate the contact state of the articular disc and condyle, the contact state of the temporal bone (such as separation and compression) and sliding and friction of the articular disc relative to the articular surface in the functional state. Effects of a lower friction coefficient of a joint on stress distribution in TMJ remain unclear [17]. According to Tanaka et al. [15, 18] the friction coefficient of normal TMJ is 0.001. When a disc is displaced, the quality and quantity of synovial fluid changes, which leads to an increase in the friction coefficient. Taking these changes into account, the friction coefficient of the side with anterior displacement of the joint disc was set to 0.001 0. 3 and 0. 4, respectively.

### Statistical analysis

All values are presented as the mean ± standard deviation (SD). Differences between the experimental and control groups were evaluated by using Student’s *t*-test. Values were determined to be significant at *P<0.05, **P<0.01, and ***P < 0.001.

## Results

**Fig. 1:**
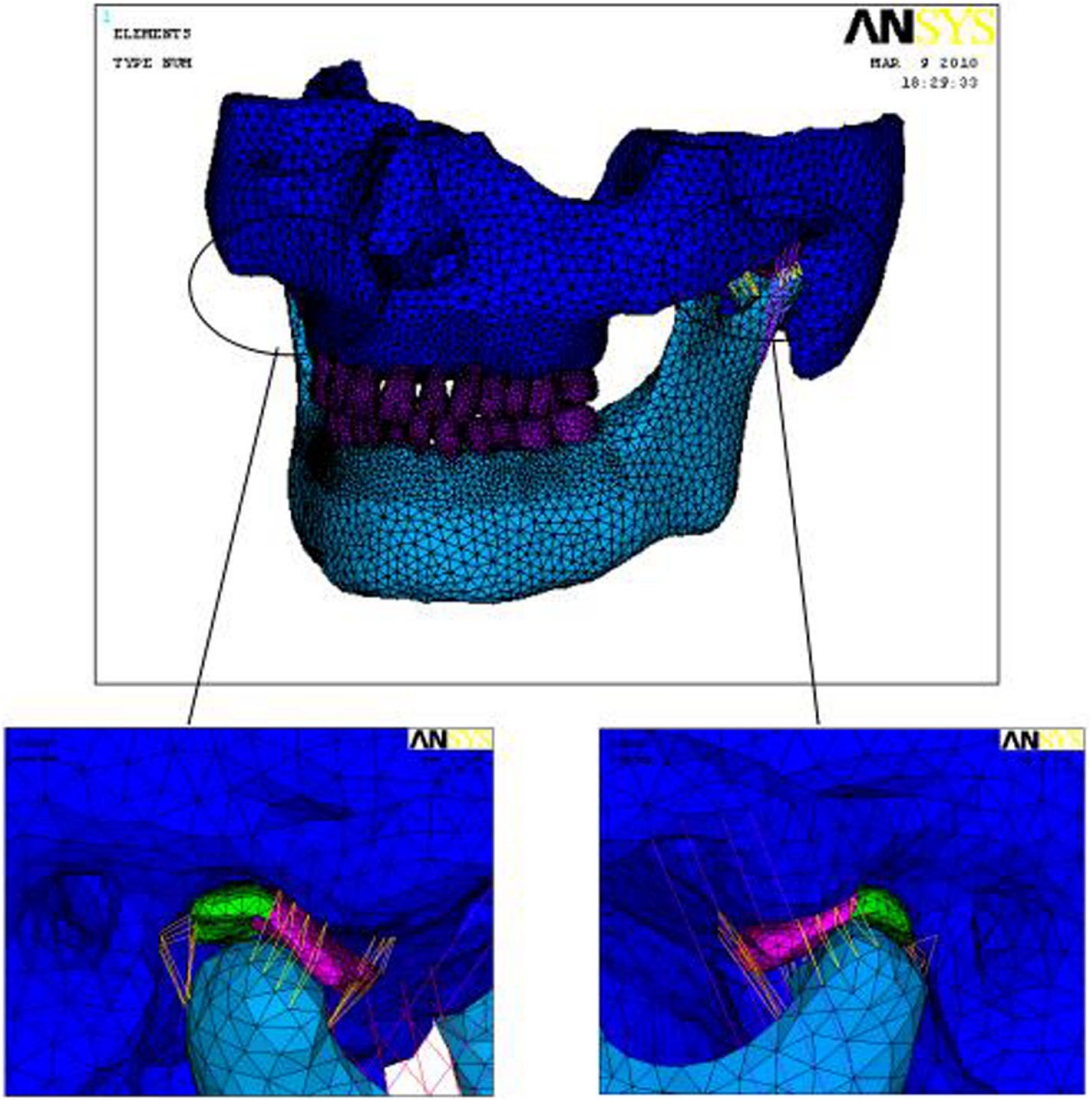
3D FEM of a normal TMJ system

**Fig. 2:**
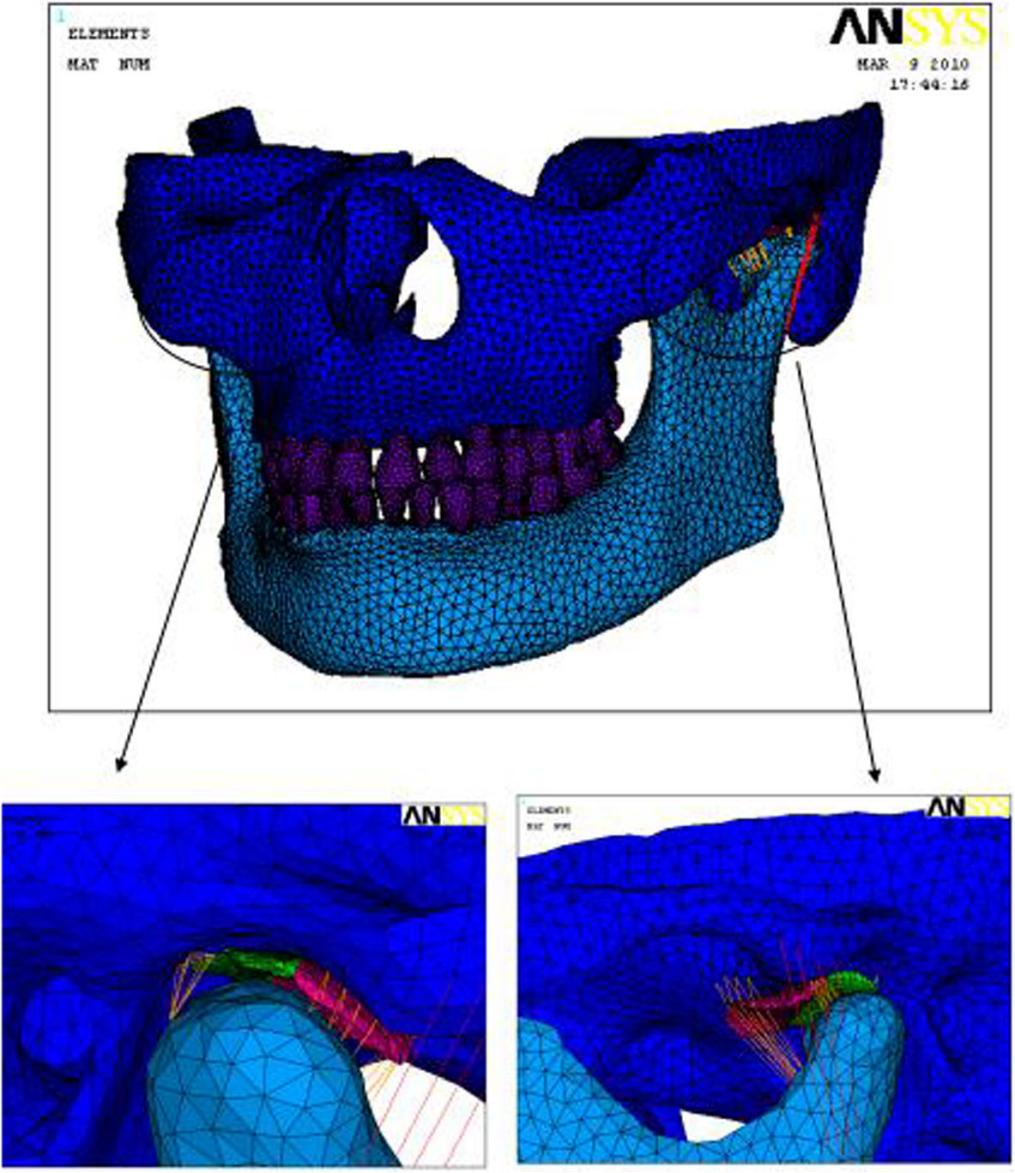
3D FEM of unilateral irreducible disc displacement

A 3D finite element model (FEM) of the normal TMJ system was established with 3891 nodes, 184,412 solid elements, 120 cable elements (Link10), 1897 contact elements and 1176 target units. On the other hand, a 3D FEM of anterior disc displacement of unilateral TMJ was established with 49,763 nodes, 237,167 solid units, 120 cable elements (Link10), 2082 contact units (Conta174) and 1812 target units (target 170) (Figs. 1 and 2).

**Fig. 3:**
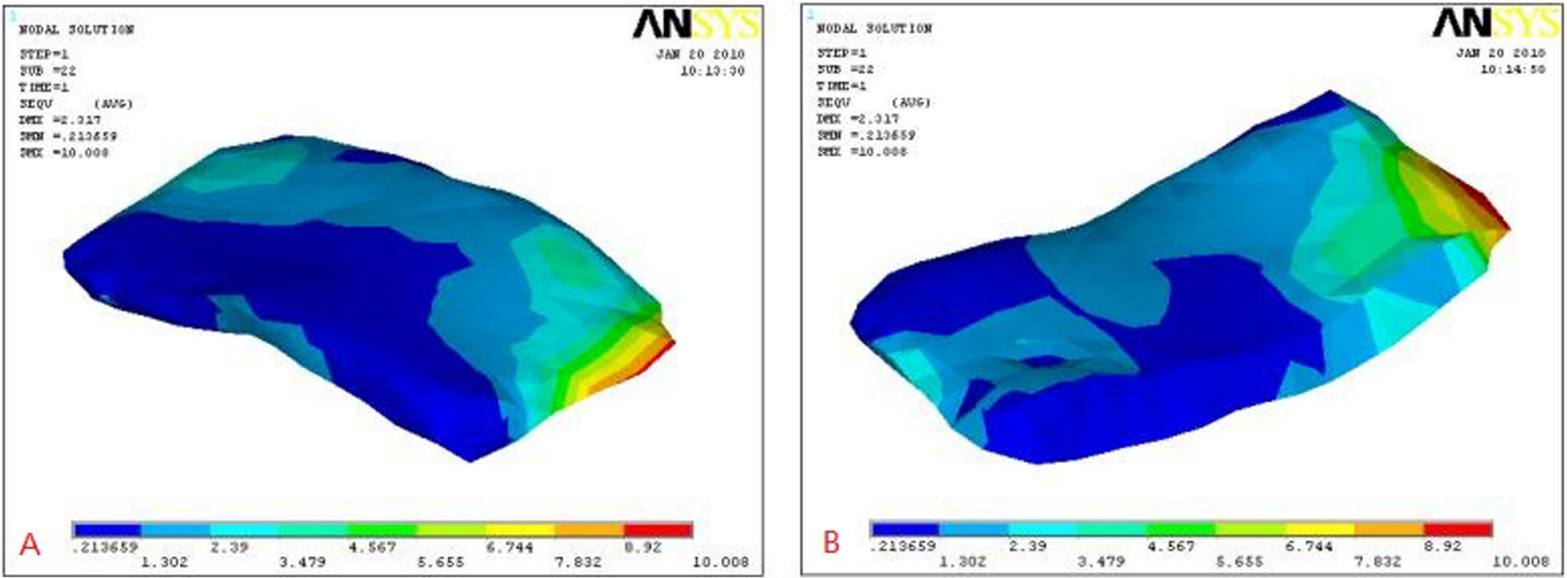
Stress distribution in a normal TMJ model: **a** upper surface, **b** lower surface. Different color represents corresponding stress

**Fig. 4:**
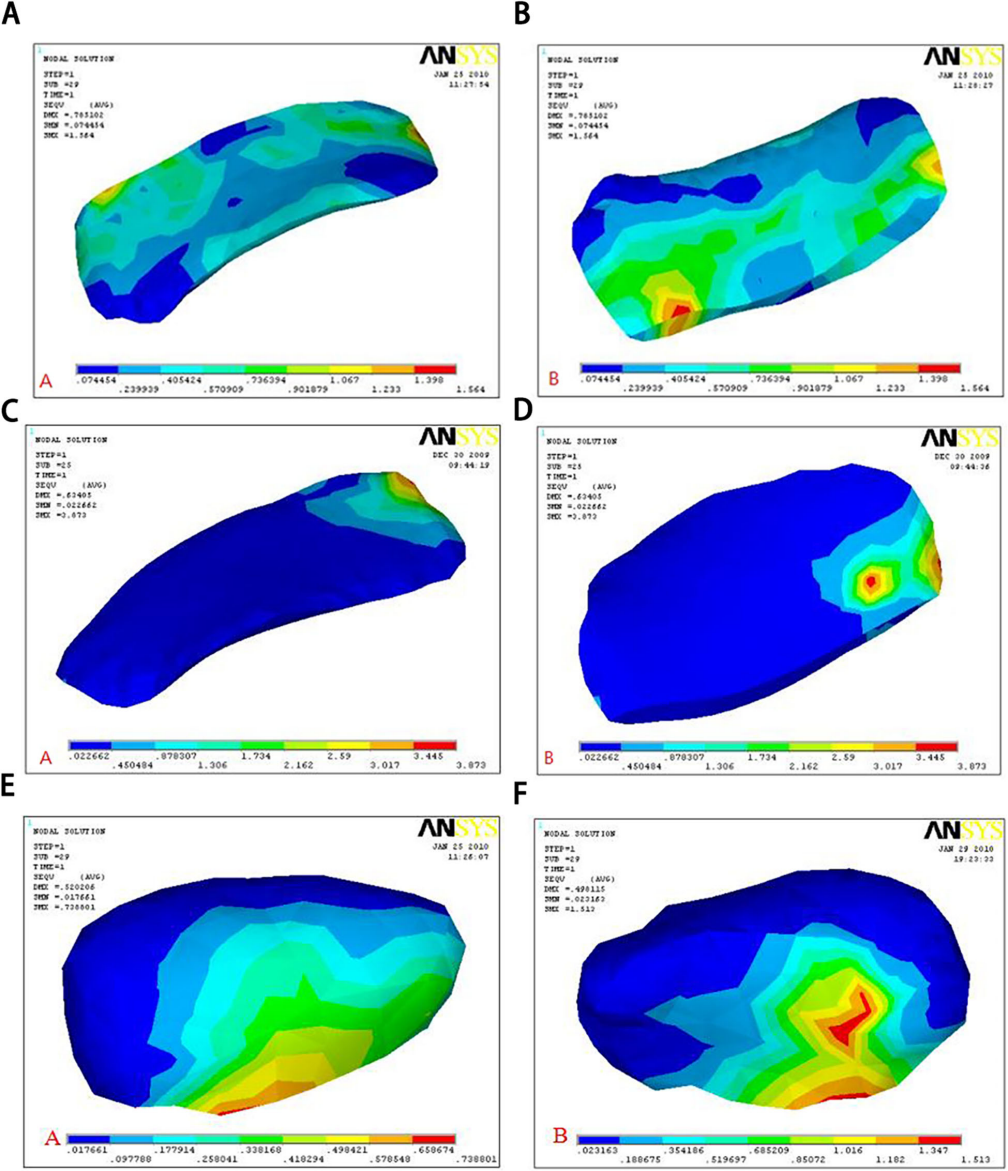
Stress distribution of the normal side disc in a pathological model: **a** upper surface, **b** lower surface. Red area is the main loading zone (C-D) Stress distribution in an anteriorly displaced lateral disc: **c** upper surface, **d** lower surface; f = 0.001. Red area denotes the main loading zone (E-F) Stress distribution on condylar of ID model: **e** normal side, **f** displaced side

Results showed that maximum stress of the normal lateral articular disc in the normal and pathological models appeared in the lateral part of the middle band. In the normal model, stress distribution was more uniform and the joint disc and the condyle were also subjected to higher load at the junction of the articular disc and the condyle (Fig. 3). On the other hand, in the pathological model, stress concentration was observed during anterior displacement of the articular disc (Fig. 4).

**Fig. 5:**
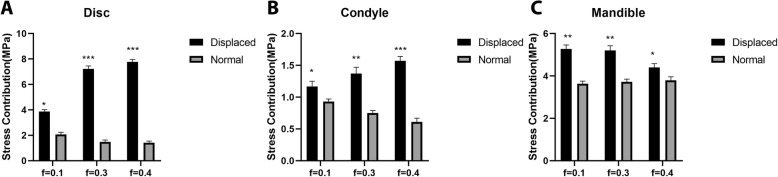
Peak values of stress on surfaces of the articular disc and condyle (MPa). **a** Stress distribution of the disc; **b** stress distribution of the condyle; **c** stress distribution of the mandible

When the friction coefficient of the side with anterior displacement increased, stress on the disc, condyle and mandible of the opposite side increased. Simultaneously, stress values of the disc, condyle and mandible were higher than those of the normal lateral joint (Table 2, Fig. 5).

**Table 2:** Peak values of stress on surfaces of the articular disc and condyle (MPa)

Position		f = 0.001	f = 0.3	f = 0.4
Disc	displaced	3.87 ± 0.15	7.23 ± 0.22	7.77 ± 0.19
	normal	2.07 ± 0.17	1.49 ± 0.14	1.41 ± 0.14
Condyle	displaced	1.17 ± 0.08	1.37 ± 0.10	1.57 ± 0.07
	normal	0.93 ± 0.04	0.75 ± 0.04	0.61 ± 0.06
Mandible	displaced	5.27 ± 0.19	5.2 ± 0.22	4.4 ± 0.18
	normal	3.63 ± 0.13	3.72 ± 0.11	3.79 ± 0.17

## Discussion

Owing to difficulties associated with direct in vivo measurements [19], computational FEM has been employed to predict the mechanochemical environment inside the TMJ under load [20, 21]. For the TMJ disc, previous FEA predicted mechanical stress distribution, fluid pressurization, and disc lubrication using elastic, nonlinear viscoelastic models [22–24] or poroelastic or biphasic mixture models [25, 26]. In patients with chronic unilateral mastication and relapse, FEA for postoperative treatment strategy such as occlusal splint would also help to predict complications after the treatment [27]. However, the impact of joint loading on the TMJ disc in patients with TMD remains unknown. As such, the study objective was to predict the force distribution in unloaded and loaded TMJ discs by using computational modelling approaches. Specifically, with subject-specific FE models developed to predict 3D force distribution in human TMJ discs with different friction coefficient.

In this study, TMJ was scanned using high-resolution CT and MRI in vivo. Advantages of CT and MRI were used to establish the joint fossa, maxilla and mandible, which included the articular fossa, maxilla and mandible. A 3D digital model of the entire dentition and the articular disc was created. All masticatory muscles, ligaments and adhesions in TMJ were simulated.

The disc is located between the condyle and the temporal bone. It functions as a cushion for stress in the joint. TMD affect stress distribution and interactions between structures in the TMJ to a certain extent, which has a negative impact on the structure and function of the joint. In Arnett and Gunson’s study, the compressive stresses were mainly loaded on the front of the condyles, causing pathological stress distribution and resorption of the condyle [28]. In this study, stress of the normal disc was concentrated in the lateral part of the middle band, and stress distribution was more uniform. However, when anterior displacement of the joint disc occurred, stress concentration was noted in the middle band of the joint disc. Such high stresses tend to lead to thinning or perforation of the joint disc. Tanaka et al. also reported that anterior displacement of the disc resulted in increase of compressive and shear stresses of the articular disc during median occlusion, which could easily cause disc thinning and perforation [29]. Moreover, Pérez-Palomar et al .[14] conducted a finite element study of the TMJ system with anterior disc displacement and found that the pressure and shear force in the posterior disc zone after anterior disc displacement of TMJ were higher than those observed in normal TMJs.

The articular capsule is lined by the synovium, and the sub-intimal layer is rich in blood vessels. It primarily provides a liquid environment for the articular surface and functions as a lubricating agent. By changing the friction coefficient of the disc of the affected side, we observed peak stress changes of the disc, condyle, and mandible and found that when the friction coefficient of the disc on the displaced side increased, increased disc friction could be observed in the corresponding side as well. Stress increased in the condyle and mandible of TMD patients, which was very similar to the results of a previous study [14]. Simultaneously, stress values of the disc, condyle, and mandible were higher than those of normal joints. According to a previous research study [30], the contact stress in the disk at the non-deviated side of patients with mandibular asymmetry in the intercuspal position (ICP) was reported to reach 2.66 MPa. Mongini et al. [31] believed that anterior disc displacement may result in flattening of the anterior oblique plane of the condyle, which is consistent with our findings that increase of the friction coefficient after anterior displacement of the articular disc leads to increase in stress on the anterior oblique plane of the condyle. NitZna et al. showed that TMJ lesions were related to abnormal position of the articular disc and increase of friction coefficient [32]. This study further proved that TMJ disease is closely related to its stress distribution. Hence, prevention and treatment of TMJ disease can be achieved by maintaining interactions among structures in normal TMJ.

Our study has some limitations. The sample size is small and heterogenous. We aim to increase the sample size of the study in future to validate these results and obtain more precise results. It is known that biomechanical models of the human TMJ are not perfect, rather they are based on a number of assumptions and simplifications. More specifically, biomechanical model could not mimic the influence of different cytokines such as IL-4, IL-6, and IL-12 [33–35]. In future studies, we aim to determine the nutrient environment in TMJ discs by using combined experimental and computational modelling approaches.

## Conclusion

3D FEMs including maxilla, articular fossa, mandible, total dentition and disc displacement of TMJ were established. At any angle, the mesh division was even and flat, and coordination between the meshes was good. Such models can directly display spatial relationships among the articular disc, mandible, articular fossa and other structures. In addition, these models can simulate various occlusal states such as the centric occlusion and forward and lateral occlusal relationships. Stress of normal TMJ disc is concentrated in the lateral part of the middle and middle zone. Stress distribution is more uniform, and stress concentration occurs in the middle zone of the joint disc before disc displacement, which can easily cause thinning or perforation of the plate. Per our research, increasing the friction coefficient between the disc and the condyle will lead to an increase in force in the TMJ region. Stress in the TMJ region with anterior displacement is greater than that in the undisplaced side, suggesting that the mechanical environment of TMJ plays an important role in the normal physiological function and formation and outcomes of TMDs.

## Data Availability

The datasets used and/or analysed during the current study available from the corresponding author on reasonable request.

## Abbreviations

TMJ: Temporomandibular joint
ID: Intra-articular disorders
FEA: Finite elements analysis, finite elements model
TMD: TMJ disorder
